# Validation of Adhesive Single-Lead ECG Device Compared with Holter Monitoring among Non-Atrial Fibrillation Patients

**DOI:** 10.3390/s21093122

**Published:** 2021-04-30

**Authors:** Soonil Kwon, So-Ryoung Lee, Eue-Keun Choi, Hyo-Jeong Ahn, Hee-Seok Song, Young-Shin Lee, Seil Oh

**Affiliations:** 1 Department of Internal Medicine, Seoul National University Hospital, Seoul 03080, Korea; david.soonil.kwon@gmail.com (S.K.); minerva1368@gmail.com (S.-R.L.); hyojeong8951@gmail.com (H.-J.A.); seil@snu.ac.kr (S.O.); 2 Department of Internal Medicine, College of Medicine, Seoul National University, Seoul 03080, Korea; 3 Seers Technology Co., Ltd., Seongnam-si 13558, Korea; sam.song@seerstech.com (H.-S.S.); luke.lee@seerstech.com (Y.-S.L.)

**Keywords:** cardiac arrhythmia, electrocardiogram, wearable device

## Abstract

There are few reports on head-to-head comparisons of electrocardiogram (ECG) monitoring between adhesive single-lead and Holter devices for arrhythmias other than atrial fibrillation (AF). This study aimed to compare 24 h ECG monitoring between the two devices in patients with general arrhythmia. Twenty-nine non-AF patients with a workup of pre-diagnosed arrhythmias or suspicious arrhythmic episodes were evaluated. Each participant wore both devices simultaneously, and the cardiac rhythm was monitored for 24 h. Selective ECG parameters were compared between the two devices. Two cardiologists independently compared the diagnoses of each device. The two most frequent monitoring indications were workup of premature atrial contractions (41.4%) and suspicious arrhythmia-related symptoms (37.9%). The single-lead device had a higher noise burden than the Holter device (0.04 ± 0.05% vs. 0.01 ± 0.01%, *p* = 0.024). The number of total QRS complexes, ventricular ectopic beats, and supraventricular ectopic beats showed an excellent degree of agreement between the two devices (intraclass correlation coefficients = 0.991, 1.000, and 0.987, respectively). In addition, the minimum/average/maximum heart rates showed an excellent degree of agreement. The two cardiologists made coherent diagnoses for all 29 participants using both monitoring methods. In conclusion, the single-lead adhesive device could be an acceptable alternative for ambulatory ECG monitoring in patients with general arrhythmia.

## 1. Introduction

Ambulatory electrocardiogram (ECG) monitoring is a useful and essential test to detect paroxysmal arrhythmias in patients with intermittent symptoms [1]. The Holter monitoring test is a representative and widely used ambulatory ECG recording test that can record ECG over 24–72 h with multiple leads. Holter monitoring can detect paroxysmal arrhythmias if the snapshot of the 12-lead ECG fails to document arrhythmic episodes [2,3]. It may be uncomfortable to attach multiple sticky electrodes to the chest and carry an ECG recording device connected by wires. However, a more extended period of monitoring is required if the arrhythmia occurs rarely [4,5,6,7]. For rarely occurring arrhythmic episodes, an implantable loop recorder can be used to monitor ECG for up to a couple of years [8,9,10]. However, invasive implantation procedures and relatively high medical expenses prohibit its convenient and broader use [11].

Compared with Holter monitoring or a loop recorder, more convenient noninvasive ECG recording devices have been developed recently with the advancement of wearable technology [12,13,14,15]. Single-lead ECG monitoring has been a favored method to record ECG with handheld or wearable devices that may replace Holter monitoring [16,17,18,19]. An adhesive patch-type ECG monitoring device is easier to use and more capable of recording ECG for an extended period than Holter monitoring [19]. However, such a device has a common disadvantage, in that it can only record the ECG along a single vector. Therefore, a single-lead ECG monitoring device may less accurately measure cardiac electrical activity with a low amplitude, such as P-waves. This issue may become crucial when discriminating cardiac arrhythmias other than atrial fibrillation (AF) because it depends on the appropriate identification of P-waves. There are few reports on head-to-head comparisons of ECG monitoring between an adhesive single-lead device and Holter monitoring among patients with cardiac arrhythmias other than AF. To improve the medical utility of a single-lead ECG device, the diagnostic characteristics of the device should be validated for various cardiac arrhythmias. One of the main hurdles of using a single-lead ECG device in actual clinical practice is a lack of sufficient validation data of a newer device than the previous gold-standard method (i.e., Holter monitoring) [20]. Although there have been several previous studies about diagnostic performance for AF detection using single-lead ECG devices [21,22], a validation study for various arrhythmia other than AF was not sufficient. This study aimed to compare 24 h ECG monitoring between an adhesive single-lead device and Holter monitoring in patients with suspicious arrhythmic episodes other than AF.

## 2. Materials and Methods

### 2.1. Study Design and Population

This was a prospective, single-center cohort study conducted at a tertiary hospital. The study consecutively included 30 participants who visited the cardiology outpatient clinic of Seoul National University Hospital from August 2020 to October 2020. Participants who were indicated for 24 h Holter monitoring for a workup of pre-diagnosed arrhythmias or suspicious arrhythmic episodes were included in the study with informed consent. To validate the diagnostic performance of the adhesive single-lead ECG device among non-AF patients, we excluded those diagnosed with AF prior to or during the study. All the study participants enrolled the study after their informed consent, and the study protocol was approved by the Seoul National University Hospital Institutional Review Board and adhered to the Declaration of Helsinki, revised in 2013 (IRB No: H-2006-224-1138).

### 2.2. Ambulatory ECG Monitoring Using an Adhesive Single-Lead ECG and Holter Monitoring

**Figure 1:**
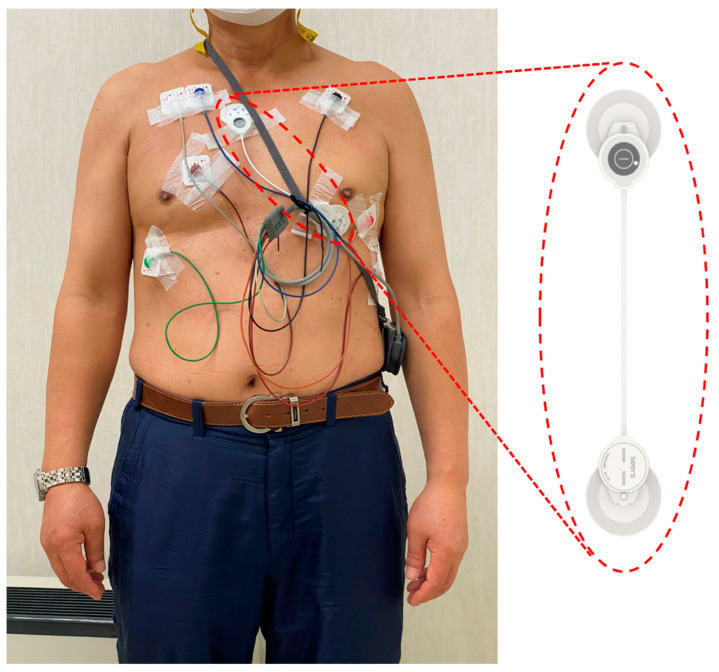
The measurement setting for ECG monitoring of a study participant. The left panel shows a participant wearing a Holter monitoring and an adhesive single-lead ECG monitoring device (mobiCARE-MC100) simultaneously. The right panel shows the product appearance of mobiCARE-MC100. Abbreviation: ECG, electrocardiogram.

At study enrollment, each participant was instructed on how to use both, the adhesive patch-type single-lead ECG monitoring device (mobiCARE-MC100, Seers Technology, Seongnam-si, Gyeonggi-do, Republic of Korea) and the Holter monitoring device (SEER Light, GE Healthcare, Chicago, IL, USA). For Holter monitoring, the study participants attached three sticky electrodes to the chest to measure leads I, II, and III. They were also required to carry the handheld recording device for 24 h. The participants wore the single-lead ECG and Holter monitoring devices simultaneously over the precordium (Figure 1). The MC-100 has two adhesive electrodes separated by 120 mm and records a single-lead ECG with a sampling rate of 256 Hz (Table 1). It operates with a replaceable battery lasting at least 72 h and transfers the ECG recording data to the preinstalled smartphone application via Bluetooth connectivity in real time. In this study, the MC-100 was patched 45° from the inter-nipple line to record lead II signals at the precordium. This position conformed to the optimal position for wearable single-lead ECG devices [23]. Detailed specifications of MC-100 are described in Table 1.

Cardiac rhythm was monitored simultaneously using both devices for 24 h. If a participant had symptoms associated with arrhythmia during monitoring, the participant recorded the event using both devices. In the case of MC-100, the event could be recorded with the preinstalled smartphone application. The ECG data acquired with the MC-100 were collected primarily from the user’s smartphone and then wirelessly transferred to a designated server for research purposes. All data stored in the server were anonymized to protect the participants’ privacy. The ECG data from the Holter monitoring device were stored in a memory within the device and then extracted to the physician’s computer via a network cable. After the 24 h monitoring, the participant returned both the devices at the clinic, and a self-report questionnaire of using the MC-100 was conducted.

### 2.3. Parameters of ECG Monitoring

To compare the diagnostic performance between single-lead ECG and Holter monitoring devices, we measured the following ECG parameters and compared them between the devices: the proportion of noise (i.e., non-analyzable noisy signals or loss of Bluetooth connection for the case of the MC-100), number of total QRS complexes/ventricular ectopic beats (VEBs)/supraventricular ectopic beats (SVEBs), burdens of VEBs and SVEBs, minimum/average/maximum heart rates, and maximum RR interval. The Holter monitoring device (SEER Light, GE Healthcare, Chicago, IL, USA) and the MC-100 recorded 24 h raw ECG data with their own software. The three cardiologists (S.K., S.-R.L., and E.-K.C.) reviewed all raw data and validated ECG parameters with each software. Finally, over the 30 study participants, the clinical diagnoses made with each device were compared. Two cardiologists (S.K. and S.-R.L.) independently evaluated the diagnoses. If a participant was diagnosed with AF during the study, the participant was excluded from the final analysis because this study aimed to focus on non-AF patients. In the case of diagnosing premature beats, frequent premature beats were defined when they exceeded 10% of the total beats.

### 2.4. Statistical Analysis

The baseline characteristics of the study population included demographic factors (age, sex, height, body weight, and body mass index), comorbidities (hypertension, diabetes mellitus, congestive heart failure, peripheral artery disease, venous thromboembolism, chronic kidney disease, chronic liver disease, and ischemic stroke), and indications for ECG monitoring. As an indication for ECG monitoring, arrhythmia-related symptoms included episodic dizziness, chest pain/discomfort, palpitation, and feelings of irregular heartbeat. Data are presented as mean ± SD, median (interquartile range), or n (%). For each ECG parameter, the degree of agreement between the two devices was evaluated using the intraclass correlation coefficient (ICC) with 95% confidence intervals. In this study, an ICC > 0.9 was regarded as an excellent agreement.

In addition, the Bland–Altman plot with 95% limits of agreement was used. The Bland–Altman plot is a statistical method to evaluate agreement in measurements between two methods [24]. It analyzes a pair of measurements with two different methods for the same subject. The differences are plotted against one of the two methods, which is frequently chosen as the “gold standard” method. The plot contains three lines, including the mean difference and limits of agreement (LoA) which could be calculated as the mean difference ± 1.96 SD of differences. The 95% confidence interval (CI) of LoA can also be calculated. If there is a difference within the upper or lower 95% CI limits of LoA, it can be 95% certain that the two methods agree in their measurements [25].

To compare the ECG parameters, individual’s total monitoring times, the proportion of noise signals between the two devices was compared, and a paired *t*-test or the Wilcoxon signed-rank test was performed. In all statistical analyses, *p* < 0.05 was considered statistically significant. Statistical analyses were performed using IBM SPSS Statistics for Windows, version 22.0 (IBM Corp, Armonk, NY, USA) and MedCalc for Windows, version 19.6.4 (MedCalc Software, Ostend, Belgium).

## 3. Results

From August 2020 to October 2020, 30 patients enrolled the study. Among the patients, one had AF during the study. As a result, 29 non-AF participants were finally evaluated in the study. Table 2 shows the baseline characteristics of the study population. The study population had a mean age of 55.1 ± 12.8 years and a male proportion of 48.3%. The most frequent comorbidity was hypertension (34.5%). The two most frequent ECG monitoring indications were workup of premature atrial contractions (41.4%) and suspicious arrhythmia-related symptoms (37.9%), including dizziness, chest pain/discomfort, palpitation, and feelings of irregular heartbeats.

### 3.1. Comparisons of ECG Monitoring Parameters between the MC-100 and Holter

All study participants completed both Holter and single-lead ECG monitoring during the study. Comparisons of ECG monitoring between the MC-100 and Holter devices are presented in Table 3. For the MC-100, the percentage of Bluetooth disconnection that occurred during the entire monitoring period for each study participant was median 0.39% with an interquartile range of 0.13–2.83%. We performed Holter and single-lead ECG monitoring during the patients’ daily living activities. In this study, the noise included various signal artifacts originating from body motions or poor electrode contacts to skin. Both monitoring methods had noise sections of less than 0.1% of the total monitoring period. The MC-100 showed a negligible amount of noise sections (0.04 ± 0.05% of total recording time), but which was significantly higher than the 24 h ECG monitoring (0.04 ± 0.05% vs. 0.01 ± 0.01%, *p* = 0.024).

**Figure 2:**
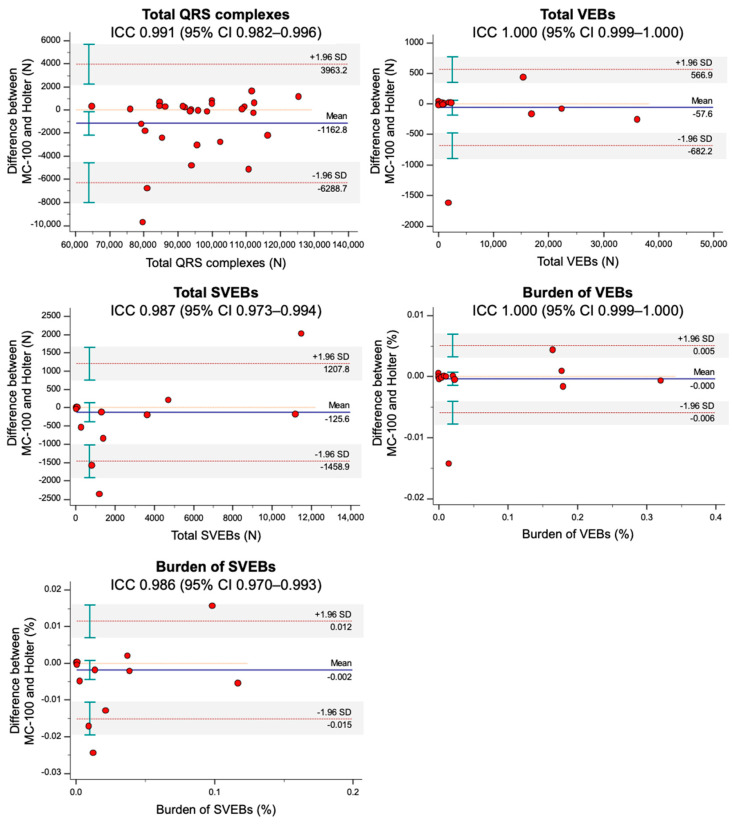
The Bland–Altman plots for ECG parameters, including total QRS complexes/VEBs/ SVEBs, and burdens of VEBs/SVEBs. The plots drew with 95% CI of limits of agreement and 95% CI of mean difference. The mobiCARE-MC100 served as a reference. Abbreviations: CI, confidence interval; ECG, electrocardiogram; ICC, intraclass correlation coefficient; SD, standard deviation; SVEB, supraventricular ectopic beat; VEB, ventricular ectopic beat.

**Figure 3:**
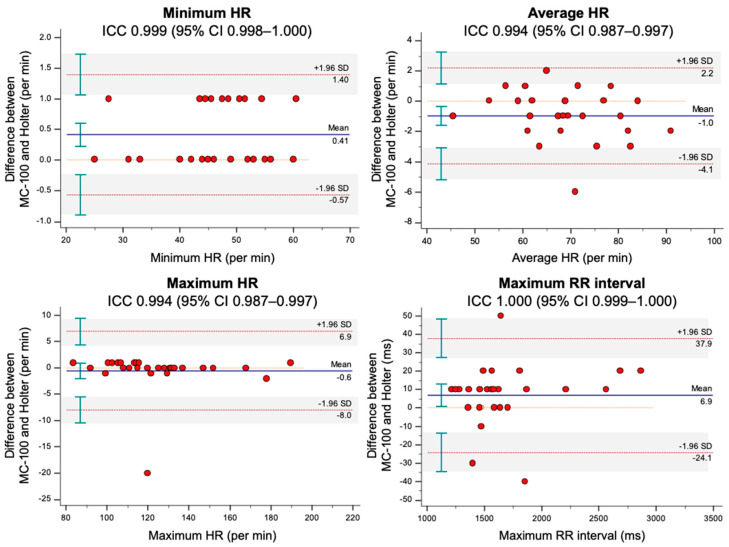
The Bland–Altman plots for ECG parameters, including minimum/average/maximum HR and maximum RR interval. The plots drew with 95% CI of limits of agreement and 95% CI of mean difference. The mobiCARE-MC100 served as a reference. Abbreviations: CI, confidence interval; ECG, electrocardiogram; HR, heart rate; ICC, intraclass correlation coefficient; SD, standard deviation.

The total number of QRS complexes/VEBs/SVEBs and the burdens of VEBs/SVEBs showed an excellent degree of agreement between the two monitoring methods (ICC = 0.991, 1.000, 0.987, 1.000, and 0.986, respectively) (Table 3). In addition, the minimum/average/maximum heart rates and maximum RR interval showed an excellent degree of agreement (ICC = 0.999, 0.994, 0.994, and 1.000). The Bland–Altman plots for the ECG parameters are presented in Figure 2 and Figure 3. Across the ECG parameters, there were no significant errors depending on the magnitudes of the measurements. In addition, regardless of the ECG parameters, most participants were measured within the limits of agreement by both monitoring methods.

Regarding the number of total QRS complexes, the MC-100 showed lower QRS complexes than the Holter device (94,910 ± 14,510 vs. 96,073 ± 13,922, *p* = 0.024), which could be related to the noise section or Bluetooth connection loss. There were no significant differences in the total number of VEBs and SVEBs between the two monitoring methods (*p* = 0.984 and 0.459, respectively). In addition, the burdens of VEBs or SVEBs were not significantly different between the two monitoring methods (*p* = 0.648 and 0.370, respectively). For the maximum heart rate, there was no significant difference between the MC-100 and Holter monitoring (123.3 ± 24.5 vs. 123.9 ± 24.7 beats/min, *p* = 0.442). There were minute but significant differences in average and minimum heart rates (69.7 ± 10.5 vs. 68.7 ± 10.1 beats/min and 46.3 ± 8.7 vs. 45.9 ± 8.6 beats/min, *p* = 0.003 and *p* < 0.001, for the MC-100 and Holter monitoring, respectively).

### 3.2. Comparison of the Clinical Diagnoses Based on Each Device between the Two Monitoring Methods

The two cardiologists independently read the ECG using the MC-100 and Holter monitoring and made coherent diagnoses for all 29 participants (Table 4): paroxysmal atrial tachycardia (6/29, 20.7%), frequent premature ventricular complexes (4/29, 13.3%), frequent premature atrial complexes (PACs) (2/29 [6.9%] for Holter monitoring and 1/29 (3.4%) for MC-100, respectively), and non-sustained ventricular tachycardia (2/29, 6.9%). Second-degree atrioventricular block of Mobitz type 1, and sick sinus syndrome were observed for the rest.

### 3.3. Self-Reported Questionnaire on Using the MC-100

A summary of the self-reported questionnaire using the MC-100 is presented in Table 5. Most participants were satisfied with using the device and application (82.8% and 79.3% with “much satisfied” or “better than Holter,” respectively). Most participants did not feel discomfort with the device, and only one participant had skin problems. Among the total study population, three (10.3%) and nine (31.0%) participants felt discomfort with the device during sleep and activity, respectively. Seventeen (58.6%) participants received the alarms due to disconnection episodes between the device and the smartphone.

## 4. Discussion

This study compared 24 h ECG monitoring between Holter and the adhesive single-lead patch-type device among patients with general arrhythmia. Our principal findings are as follows: (i) there were excellent agreements in ECG parameters including the total QRS complexes/VEBs/SVEBs, burdens of VEBs/SVEBs, minimum/average/maximum heart rates, and maximum RR intervals; (ii) there were no significant differences in ECG parameters including total VEBs/SVEBs, burdens of VEBs/SVEBs, and maximum heart rates; (iii) the MC-100 had a higher proportion of noise sections than Holter; however, both devices showed negligible proportions of noise sections; (iv) clinical diagnoses were coherent regardless of the monitoring methods; and (v) most participants felt comfortable using the adhesive single-lead ECG monitoring device for 24 h.

### 4.1. Current Status of Adhesive Single-Lead ECG Monitoring Device

With the recent development of wearable and smartphone technology, various types of portable ECG monitoring devices have been reported [26,27]. Among them, single-lead monitoring has been the most commonly used method to measure ECG [28]. There have been adhesive patch-type devices by attaching electrodes to the precordium or handheld-type devices that can be carried by a patient [26,27,29,30]. Patch-type devices have an advantage of being able to monitor ECG signals continuously once a patient wears them. Depending on the products, patch-type devices can measure ECG for up to a couple of weeks and transmit ECG data to the patient’s smartphone or store the data in their memory [19,31,32]. Compared with the conventional Holter test, monitoring ECG only up to a single lead may be a disadvantage of most patch-type devices. However, patients may prefer a patch-type device to the Holter test because it is more convenient to wear for an extended monitoring period [19]. Multiple studies have reported that patch-type devices could detect arrhythmic episodes as accurately as the Holter test [17,33,34]. Moreover, there was a report that screening arrhythmia using these devices would be cost-effective [35]. As a result, a patch-type device has become a useful diagnostic tool for cardiac arrhythmia and has gained attention as an alternative to the Holter test.

### 4.2. Comparisons of the MC-100 and Holter Monitoring

Table 6 summarizes the comparison between the MC-100 and Holter monitoring. Compared to Holter monitoring, the MC-100 can monitor ECG data in real-time with the user’s smartphone. The application should be installed on the user’s smartphone, and the MC-100 must be connected with it via Bluetooth to enable real-time monitoring. Therefore, it has advantages in real-time ECG monitoring but potentially has a risk of data loss due to Bluetooth disconnection. It has a comparable monitoring period (up to 3 days) compared with Holter monitoring. However, a user may easily replace the battery and keep using the device. The MC-100 has an advantage in light product weight (9 g). It uses the same replaceable ECG electrodes as Holter’s electrodes. The device is easily reusable because it uses replaceable electrodes and a coin cell battery. In summary, we postulate that the MC-100 is likely to be beneficial when confirming whether the patient’s symptom is associated with intermittent arrhythmia because it provides real-time ECG monitoring. Moreover, better compliance of using the device is expected with the MC-100 due to its light weight and a compact size.

### 4.3. Differences in ECG Measurements between the MC-100 and Holter

In this study, the MC-100 had longer monitoring by 5 min than Holter owing to the order of attachment of the device. However, the total number of QRS complexes was smaller in MC-100. The difference could be explained by (i) a higher proportion of noise sections with the MC-100 than with Holter and (ii) an additional monitoring loss due to Bluetooth disconnection between the MC-100 and user’s smartphone. Both the MC-100 and Holter computed the heart rate as an average rate over the six most recent RR intervals. Although there were statistically significant differences in the minimum and average heart rates between the two monitoring methods, the magnitudes of the differences were only up to 1 beat/min. The loss of Bluetooth connection for more than 1% of the total monitoring period occurred in 11 out of 29 patients, and the primary reason was the increased distance between the device and smartphone. None of the participants had detached episode(s) with the device, and 17 participants received alarms from their smartphones for device disconnection (Table 5).

### 4.4. Limitations in Single-Lead ECG Monitoring

**Figure 4:**
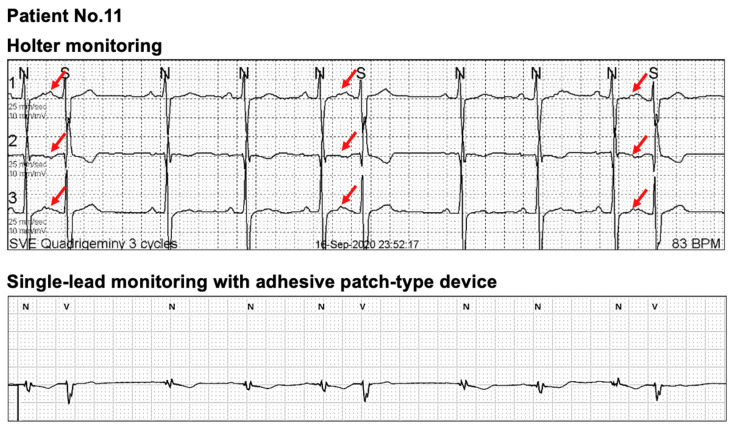
An exemplary case of reporting more VEBs with a single-lead ECG monitoring than with Holter monitoring. The participant had PACs with aberrant conduction (red arrows) in Holter monitoring. However, the aberrant conduction was less clear in this participant because the P-waves are unclear in the single-lead ECG monitoring. Therefore, for the patients with P-waves of low amplitude, a compensatory pause should be considered in addition to the change in QRS morphology when judging a premature ventricular complex or aberrant conduction with the single-lead ECG monitoring. Abbreviations: ECG, electrocardiogram; PAC, premature atrial complex; VEB, ventricular ectopic beat.

**Figure 5:**
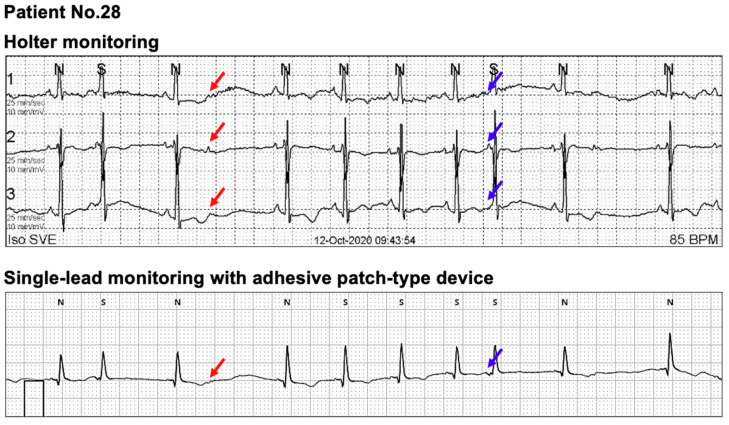
An exemplary case of reporting more SVEBs with a single-lead ECG monitoring than with Holter monitoring. In this participant, non-conducted PACs (red arrows) and conducted PACs (blue arrows) were observed in Holter monitoring, whereas P-waves were rarely visible with the single-lead monitoring. Compared to the sinus rhythm, the P-wave morphology was different in non-conducted and conducted PACs in Holter monitoring. Therefore, detecting SVEBs with single-lead monitoring mostly depends on evaluating RR intervals and QRS morphology. As a result, SVEBs may be overestimated with the single-lead monitoring than with Holter for this case. Abbreviations: ECG, electrocardiogram; PAC, premature atrial complex; SVEB, supraventricular ectopic beat.

The P-wave axis was different among the participants; therefore, some P-waves were not clearly recognizable in the single-lead ECG monitoring. In Figure 4, the participant had PACs with aberrant conduction (red arrows) during Holter monitoring. However, the aberrant conduction was less clear in this participant because the P-waves were unclear in the single-lead ECG monitoring. Therefore, for patients with P-waves of low amplitudes, a compensatory pause should be considered in addition to the change in QRS morphology when judging a premature ventricular complex or aberrant conduction with single-lead ECG monitoring. Moreover, there was a case that non-conducted PACs could not be recognized. In Figure 5, non-conducted PACs (red arrows) and PACs (blue arrows) were observed in Holter monitoring, whereas P-waves were rarely visible with single-lead monitoring. Compared with the sinus rhythm, the P-wave morphology was different in non-conducted and conducted PACs in Holter monitoring. Therefore, detecting SVEBs with single-lead monitoring mostly depends on evaluating the RR intervals and QRS morphology. As a result, SVEBs may be overestimated with single-lead monitoring than with Holter monitoring in this case.

**Figure 6:**
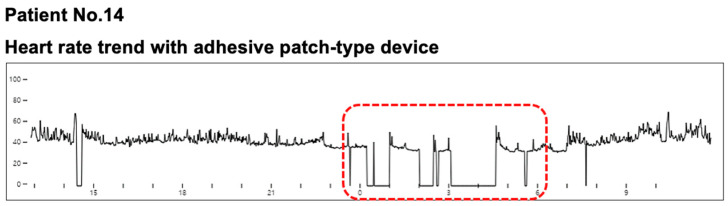
The heart rate trend with adhesive patch-type ECG monitoring device. The raw data from the outlier with significantly higher total QRS complexes with Holter monitoring showed there were significant Bluetooth disconnection periods during the nocturnal period (within the red-rectangled area). Abbreviations: ECG, electrocardiogram.

**Figure 7:**
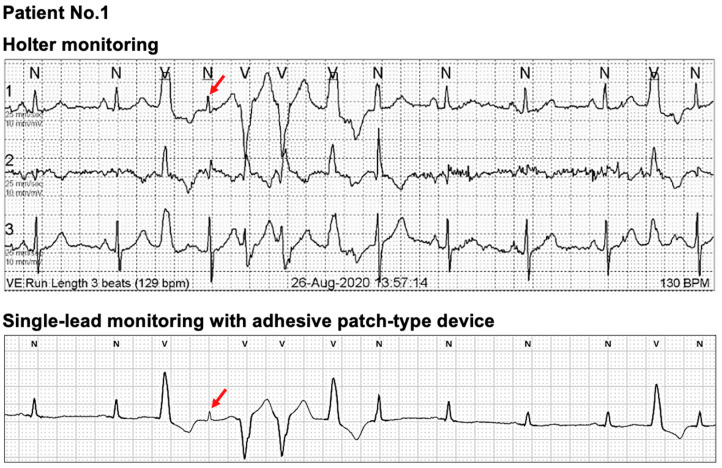
The outlier with significantly higher maximum heart rate with Holter monitoring. The amplitude of a particular QRS complex (red arrow) was lower than other complexes in the lead I, and this led to undersensing of the QRS complex with the MC-100. Such a particular undersensing led to different measurements of the maximum heart rate.

**Figure 8:**
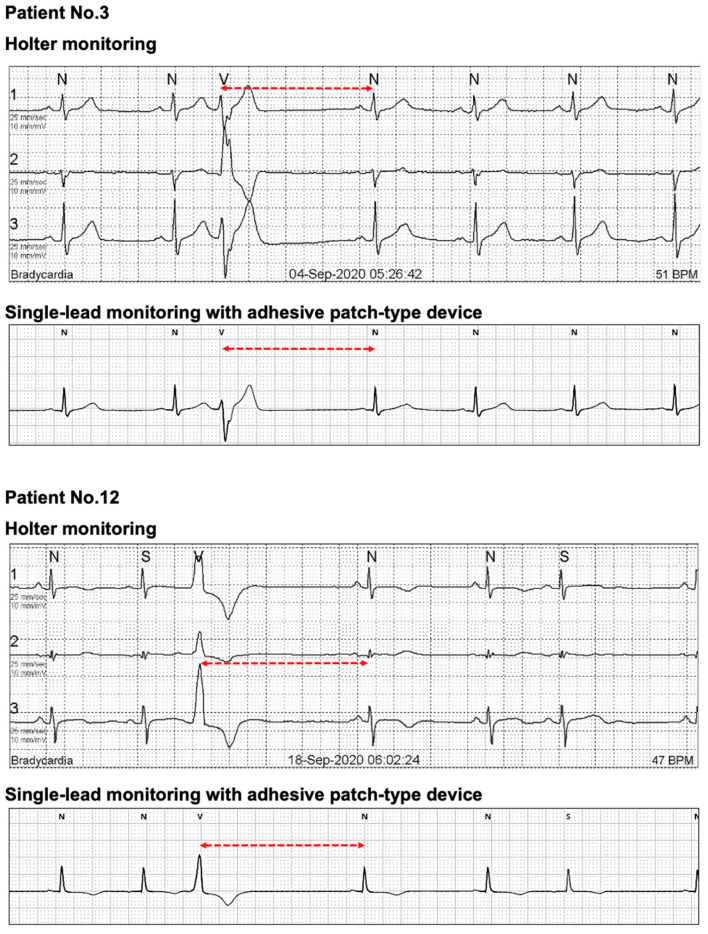
The outlier with different maximum RR intervals between the two devices. Both devices detected the same episode for their detection of the maximum RR interval. However, there was a minute difference in maximum RR intervals (red lines) between both devices (less than 50 ms). We concluded there existed minute differences in detecting R-wave peaks between both devices.

In Figure 2, one outlier with significantly higher QRS complexes with Holter monitoring than MC-100 was observed. A detailed evaluation of the patient’s raw data from the MC-100 revealed significant Bluetooth disconnection during the nocturnal period. This signal loss resulted in a significantly lower number of detected QRS complexes (Figure 6). As shown in Figure 3, there was one outlier with a significantly higher average heart rate with Holter monitoring. However, a detailed examination of both raw data of the two devices revealed no marked abnormalities. Another patient had a significantly higher maximum heart rate with Holter monitoring. We found that the amplitude of a particular QRS complex was lower than that of other complexes in lead I, and this led to the undersensing of the QRS complex with the MC-100 (Figure 7). This particular undersensing led to different measurements of the maximum heart rate. In most study participants, except for the outliers, there were still differences in heart rate measurements. However, the mean differences in minimum/average/maximum heart rates for the two devices were only up to one beat per minute or less (Figure 3). For maximum RR interval, the mean difference between the two devices was 6.9 ms (Figure 3). There were two outliers with significantly different maximum RR intervals between the two devices. Both devices detected the same episode for their detection of the maximum RR interval (Figure 8). However, there was a minute difference in maximum RR intervals (red lines) between both devices (less than 50 ms). We concluded that minute differences existed in the detection of R-wave peaks between the two devices. Minute differences in QRS morphology recorded between the two devices may lead to slightly different measures in RR intervals.

In summary, The single-lead ECG device could be an excellent alternative to Holter monitoring in most participants. However, some exceptional cases, such as the outliers mentioned previously, should be remarked when physicians plan to use the single-lead ECG device in their practices.

### 4.5. Limitations and Strengths

This study has some limitations. First, the proportion of lost signals due to Bluetooth disconnection may have affected the results of the MC-100. However, the proportion of lost connections was only 0.39%. Since most disconnections occurred when participants were away from their smartphones, this error could be minimized if the education on using the device was enhanced. If an intrinsic memory chip can store ECG signals over a short period during disconnection, such an error can be fundamentally reduced. Second, this study could not evaluate ECG parameters, including QRS axis, QRS duration, or QT interval, because these are not analyzable with Holter. Third, different algorithms to calculate heart rates or detect VEB/SVEB may affect the results between devices. We found that the differences were minimal, and the results were generally acceptable. However, there is a concern that a small difference in ECG parameters may be critical in judging the treatment plan in exceptional cases, such as deciding pacemaker implantation for bradyarrhythmia. Fourth, this study could not validate specific arrhythmia subgroups such as sick sinus syndrome, atrial flutter, AF, atrioventricular blocks, and sustained ventricular tachycardia because of the small population size. A future study with a larger population is needed to validate the single-lead ECG monitoring with the adhesive patch-type device in subgroups of particular arrhythmias. Finally, our study lacks a theoretical approach for comparisons of ECG signal characteristics between adhesive single-lead ECG device and Holter test. The distance between and positions of electrodes may affect the signal characteristics with the single-lead ECG device. There was also a lack of analysis on the optimization of electrodes’ positions with the single-lead ECG device. These contents are beyond the scope of the current study. Further studies are needed to address these issues.

Our study has the following strengths. First, we focused on patients with general cardiac arrhythmias except for AF. Up to date, most clinical studies with single-lead ECG devices focused on patients with AF. Second, albeit its importance, there are fewer reports on validating basic ECG parameters between the two methods for non-AF patients. To improve the medical utility of a single-lead ECG device, the diagnostic characteristics of the device should be validated for various cardiac arrhythmias. Third, we investigated special cases with discrepant measurements in ECG parameters between the two methods. From Figure 4, Figure 5, Figure 6, Figure 7 and Figure 8, we analyzed raw data from both devices for some outliers. As we hypothesized previously, we found poorer P-wave distinction with adhesive single-lead ECG device led to less accurate measurements in some ECG parameters (Figure 4 and Figure 5). Moreover, undersensing of QRS complexes could unexpectedly lead to less accurate heart rate measurement (Figure 7). We believe these special cases should be remarked for physicians when they use adhesive single-lead ECG devices to their patients to detect non-AF cardiac arrhythmias.

## 5. Conclusions

Compared with traditional Holter monitoring, single-lead monitoring with adhesive patch-type device has acceptable accuracy for ECG monitoring and is more convenient to use. In general, there was excellent agreement in ECG parameters and cardiologists’ diagnoses between the single-lead ECG monitoring with the adhesive patch-type device and Holter monitoring. However, interpreting single-lead monitoring may require caution when P-wave signals are less identifiable. Further studies are warranted to validate this device in clinical practice for various arrhythmia populations.

## Figures and Tables

**Table 1:** The specification of the MC-100.

Manufacturer	Seers Technology (Seongnam-si, Gyeonggi-do, Republic of Korea)
Product serial number	mobiCARE-MC100
Product appearance	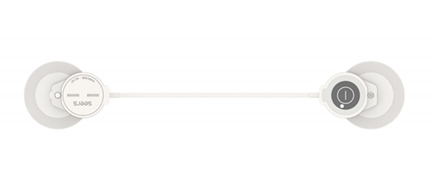
Size	Width 29 mm, length 120 mm
Weight	8.9 g
Measurements	Single-lead electrocardiogram in real-time, heart rate, movement activity
Sensors	Electrodes, accelerometers, gyroscopes
Connectivity	Bluetooth low energy
Heart rate measurement range	From 30 to 240 beat-per-minutes
Sampling rate	256 Hz
Battery	Replaceable CR2032H coin cell battery
Electrode standard	Medical standard 4.0 mm electrode snaps for electrocardiogram
Operating time	Lasting at least 72 h continuously

**Table 2:** Baseline characteristics of the study participants (total *N* = 29).

Characteristics	Value
Demographic factors	
Age, year	55.1 ± 12.8
Male, %	14 (48.3)
Height, cm	164.5 ± 6.7
Weight, kg	66.6 ± 11.1
Body mass index, kg/m^2^	24.5 ± 2.8
Comorbidity	
Hypertension	10 (34.5)
Diabetes mellitus	2 (6.9)
Congestive heart failure	1 (3.4)
Peripheral artery disease	0 (0)
Ischemic heart disease	0 (0)
Chronic kidney disease	2 (6.9)
Chronic liver disease	1 (3.4)
Ischemic stroke	1 (3.4)
Indication for ECG monitoring ^1^	
Suspicious arrhythmia-related symptoms ^2^	11 (37.9)
History of arrhythmia	20 (69.0)
Premature atrial contraction	2 (6.9)
Supraventricular tachyarrhythmia	3 (10.3)
Atrial flutter	1 (3.4)
Premature ventricular contraction	12 (41.4)
Idiopathic ventricular tachycardia	1 (3.4)
Second-degree atrioventricular block, Mobitz type 1	1 (3.4)

The data were presented as mean ± standard deviation or n (%). ^1^ One patient had both premature atrial contraction and premature ventricular contraction. One patient had both supraventricular tachyarrhythmia and premature ventricular contraction. ^2^ Arrhythmia-related symptoms included dizziness, chest pain/discomfort, palpitation, and feelings of irregular heartbeats. Abbreviations: ECG; electrocardiogram.

**Table 3:** Comparisons of ECG monitoring between Holter and adhesive single-lead ECG monitoring for the study population.

	Holter	mobiCARE-MC100	ICC (95% CI)	*p*-Value for Reliability	*p*-Value for Mean Difference
Total participants, N	29	29	-	-	-
Noise, %	0.01 ± 0.01	0.04 ± 0.05 ^1^	-	-	0.024
Total wear time, min ^2^	1403 ± 20	1408 ± 20	-	-	<0.001
Total QRS complexes, N	96,073 ± 13,922	94,910 ± 14,510	0.991 (0.982–0.996)	<0.001	0.024
Total VEBs, N	6 (1–948)	5 (1–1459)	1.000 (0.999–1.000)	<0.001	0.984
Total SVEBs, N	25 (8–93)	48 (13–1485)	0.987 (0.973–0.994)	<0.001	0.459
Burden of VEBs, %	0.01 (0–0.96)	0.01 (0–1.57)	1.000 (0.999–1.000)	<0.001	0.648
Burden of SVEBs, %	0.03 (0.01–0.10)	0.05 (0.01–1.62)	0.986 (0.970–0.993)	<0.001	0.370
Patients with frequent VEBs, N (%) ^3^	4 (13.8)	4 (13.8)	-	-	>0.999
Patients with frequent SVEBs, N (%) ^3^	2 (6.9)	1 (3.4)	-	-	0.317
Minimum HR, beats/min	45.9 ± 8.6	46.3 ± 8.7	0.999 (0.998–1.000)	<0.001	<0.001
Average HR, beats/min	68.7 ± 10.1	69.7 ± 10.5	0.994 (0.987–0.997)	<0.001	0.003
Maximum HR, beats/min	123.9 ± 24.7	123.3 ± 24.5	0.994 (0.987–0.997)	<0.001	0.442
Maximum RR interval, ms	1560 (1460–1755)	1570 (1465–1765)	1.000 (0.999–1.000)	<0.001	<0.001

Data are n (%), mean ± standard deviation, or median (interquartile range). ^1^ Except for the signal loss due to Bluetooth disconnection (median 0.39% with interquartile range of 0.13–2.83%). ^2^ The total wear time is the duration between the attachment and the detachment of each device. ^3^ Patients with SVEB or VEB burdens ≥10.0%. Abbreviations: CI, confidence interval; ECG, electrocardiogram; HR, heart rate; ICC, intraclass correlation coefficient; SVEB, supraventricular ectopic beat; VEB, ventricular ectopic beat.

**Table 4:** Comparisons of diagnoses between Holter and adhesive single-lead ECG monitoring by cardiologists.

Patient No.	Holter	MC-100
Patient 1	Frequent PVC ^1^	Frequent PVC ^1^
Patient 2	Frequent PVC ^1^	Frequent PVC ^1^
Patient 3	Predominantly SR ^2^	Predominantly SR ^2^
Patient 4	PAT	PAT
Patient 5	Predominantly SR ^2^	Predominantly SR ^2^
Patient 6	Predominantly SR ^2^	Predominantly SR ^2^
Patient 7	Predominantly SR ^2^	Predominantly SR ^2^
Patient 8	PAT	PAT
Patient 9	Predominantly SR ^2^	Predominantly SR ^2^
Patient 10	Predominantly SR ^2^	Predominantly SR ^2^
Patient 11	PAT, frequent PAC ^1^	PAT
Patient 12	Frequent PVC ^1^	Frequent PVC ^1^
Patient 13	Predominantly SR ^2^	Predominantly SR ^2^
Patient 14	Predominantly SR ^2^	Predominantly SR ^2^
Patient 15	Predominantly SR ^2^	Predominantly SR^2^
Patient 16	Second-degree AVB (type 1), NSVT	Second-degree AVB (type 1), NSVT
Patient 17	Predominantly SR ^2^	Predominantly SR ^2^
Patient 18	Frequent PVC ^1^, NSVT	Frequent PVC ^1^, NSVT
Patient 19	Predominantly SR ^2^	Predominantly SR ^2^
Patient 20	Predominantly SR ^2^	Predominantly SR^2^
Patient 21	PAT	PAT
Patient 22	Predominantly SR ^2^	Predominantly SR ^2^
Patient 23	Predominantly SR ^2^	Predominantly SR ^2^
Patient 24	Predominantly SR ^2^	Predominantly SR ^2^
Patient 25	SSS	SSS
Patient 26	Predominantly SR ^2^	Predominantly SR ^2^
Patient 27	Predominantly SR ^2^	Predominantly SR ^2^
Patient 28	PAT, frequent PAC ^1^	PAT, frequent PAC ^1^
Patient 29	PAT	PAT

^1^ In this study, frequent PAC or PVC was defined as a burden of supraventricular or ventricular ectopic beats ≥10% with predominantly sinus rhythm. ^2^ Predominantly SR was defined as SR with infrequent premature beats less than 10% of total beats without other types of arrhythmias. Abbreviations: AVB, atrioventricular block; ECG, electrocardiogram; NSVT, non-sustained ventricular tachycardia; PAC, premature atrial complex; PAT, paroxysmal atrial tachycardia; PSVT, paroxysmal supraventricular tachycardia; PVC, premature ventricular complex; SR, sinus rhythm; SSS, sick sinus syndrome.

**Table 5:** The self-reported questionnaire on using the MC-100 (English-translated version).

	Mean ± SD or *N* (%)
Usability of the adhesive single-lead ECG monitoring device	
Did you feel discomfort with the device? (None = 1, Minimal = 2, Some = 3, Much = 4, Very much = 5)	1.6 ± 1.0
Did you feel skin irritability with the device? (None = 1, Minimal = 2, Some = 3, Much = 4, Very much = 5)	1.8 ± 1.2
When do you most feel the discomfort of using the device?	
During sleep	3 (10.3)
During activity	9 (31.0)
During rest	0 (0)
Did you have detached episode(s) with the device?	0 (0)
Usability of the smartphone application for the monitoring device	
Did you check the application for monitoring your ECG? (None = 1, Minimal = 2, Some = 3, Much = 4, Very much = 5)	2.4 ± 1.3
Did you record an episode with the application when you had symptoms? (None = 1, Minimal = 2, Some = 3, Much = 4, Very much = 5)	1.7 ± 1.3
Was it easy to record your symptom with the application? (None = 1, Minimal = 2, Some = 3, Much = 4, Very much = 5)	2.5 ± 1.4
Did you receive alarms from the application for the device disconnection?	17 (58.6)
Overall product evaluation	
Do you satisfy with using the device? (None = 1, Minimal = 2, Some = 3, Much = 4, Very much = 5)	4.1 ± 1.0
Do you satisfy with using the application? (None = 1, Minimal = 2, Some = 3, Much = 4, Very much = 5)	4.0 ± 1.2

Abbreviations: ECG; electrocardiogram; SD, standard deviation.

**Table 6:** Brief comparisons between the MC-100 and Holter.

	MC-100	Holter
Manufacturer	Seers Technology	Variable
Monitoring period	Up to 3 days	Up to 3 days
ECG channels	1	3–12, variable
Real-time monitoring	Yes	No
Weight	9 g	Typically over 100 g, variable
Battery	Replaceable commercial coin cell battery	Mostly built-in, variable
Electrodes	Replaceable commercial ECG electrodes	Replaceable commercial ECG electrodes
Data storage	User’s smartphone with the application is necessary to monitor and store ECG data. Bluetooth connectivity is required	Built-in memory. Data transmission and processing are processed in the clinic
Associated components	Electrodes only	Electrodes, leads, symptom recorder, straps, variable

Abbreviations: ECG, electrocardiogram.

## Data Availability

The data presented in this study are available on request from the corresponding author. The data are not publicly available due to ethical reasons.

## References

[1] Ikeda T. (2021). Current use and future needs of noninvasive ambulatory electrocardiogram monitoring. Intern. Med..

[2] Shen W.-K., Sheldon R.S., Benditt D.G., Cohen M.I., Forman D.E., Goldberger Z.D., Grubb B.P., Hamdan M.H., Krahn A.D., Link M.S. (2017). 2017 ACC/AHA/HRS guideline for the evaluation and management of patients with syncope: A report of the American College of Cardiology/American Heart Association Task Force on clinical practice guidelines and the Heart Rhythm Society. Circulation.

[3] Hindricks G., Potpara T., Dagres N., Arbelo E., Bax J.J., Blomström-Lundqvist C., Boriani G., Castella M., Dan G.A., Dilaveris P.E. (2021). 2020 ESC Guidelines for the diagnosis and management of atrial fibrillation developed in collaboration with the European Association of Cardio-Thoracic Surgery (EACTS). Eur. Heart J..

[4] Busch M.C., Gross S., Alte D., Kors J.A., Völzke H., Ittermann T., Werner A., Krüger A., Busch R., Dörr M. (2017). Impact of atrial fibrillation detected by extended monitoring—A population-based cohort study. Ann. Noninvasive Electrocardiol..

[5] Quan K.J. (2019). Palpitation: Extended electrocardiogram monitoring: Which tests to use and when. Med. Clin. N. Am..

[6] Turakhia M.P., Ullal A.J., Hoang D.D., Than C.T., Miller J.D., Friday K.J., Perez M.V., Freeman J.V., Wang P.J., Heidenreich P.A. (2015). Feasibility of extended ambulatory electrocardiogram monitoring to identify silent atrial fibrillation in high-risk patients: The screening study for undiagnosed atrial fibrillation (STUDY-AF). Clin. Cardiol..

[7] Steinhubl S.R., Waalen J., Edwards A.M., Ariniello L.M., Mehta R.R., Ebner G.S., Carter C., Baca-Motes K., Felicione E., Sarich T. (2018). Effect of a home-based wearable continuous ECG monitoring patch on detection of undiagnosed atrial fibrillation: The mSToPS randomized clinical trial. JAMA.

[8] Krahn A.D., Klein G.J., Skanes A.C., Yee R. (2004). Insertable loop recorder use for detection of intermittent arrhythmias. Pacing Clin. Electrophysiol..

[9] Hindricks G., Pokushalov E., Urban L., Taborsky M., Kuck K.-H., Lebedev D., Rieger G., Pürerfellner H. (2010). Performance of a new leadless implantable cardiac monitor in detecting and quantifying atrial fibrillation: Results of the XPECT trial. Circ. Arrhythm. Electrophysiol..

[10] Brachmann J., Morillo C.A., Sanna T., Di Lazzaro V., Diener H.C., Bernstein R.A., Rymer M., Ziegler P.D., Liu S., Passman R.S. (2016). Uncovering atrial fibrillation beyond short-term monitoring in cryptogenic stroke patients: Three-year results from the cryptogenic stroke and underlying atrial fibrillation trial. Circ. Arrhythm. Electrophysiol..

[11] Chew D.S., Rennert-May E., Spackman E., Mark D.B., Exner D.V. (2020). Cost-effectiveness of extended electrocardiogram monitoring for atrial fibrillation after stroke: A systematic review. Stroke.

[12] Sana F., Isselbacher E.M., Singh J.P., Heist E.K., Pathik B., Armoundas A.A. (2020). Wearable devices for ambulatory cardiac monitoring: JACC state-of-the-art review. J. Am. Coll. Cardiol..

[13] Ip J.E. (2019). Wearable devices for cardiac rhythm diagnosis and management. JAMA.

[14] Perez M.V., Mahaffey K.W., Hedlin H., Rumsfeld J.S., Garcia A., Ferris T., Balasubramanian V., Russo A.M., Rajmane A., Cheung L. (2019). Large-scale assessment of a smartwatch to identify atrial fibrillation. N. Engl. J. Med..

[15] Hwang J., Kim J., Choi K.J., Cho M.S., Nam G.B., Kim Y.H. (2019). Assessing accuracy of wrist-worn wearable devices in measurement of paroxysmal supraventricular tachycardia heart rate. Korean Circ. J..

[16] Heckbert S.R., Austin T.R., Jensen P.N., Floyd J.S., Psaty B.M., Soliman E.Z., Kronmal R.A. (2018). Yield and consistency of arrhythmia detection with patch electrocardiographic monitoring: The multi-ethnic study of atherosclerosis. J. Electrocardiol..

[17] Nault I., André P., Plourde B., Leclerc F., Sarrazin J.-F., Philippon F., O’Hara G., Molin F., Steinberg C., Roy K. (2019). Validation of a novel single lead ambulatory ECG monitor—Cardiostat—Compared to a standard ECG Holter monitoring. J. Electrocardiol..

[18] Karaoğuz M.R., Yurtseven E., Aslan G., Deliormanlı B.G., Adıgüzel Ö.G.M., Li K.-M., Yılmaz E.N. (2019). The quality of ECG data acquisition, and diagnostic performance of a novel adhesive patch for ambulatory cardiac rhythm monitoring in arrhythmia detection. J. Electrocardiol..

[19] Barrett P.M., Komatireddy R., Haaser S., Topol S., Sheard J., Encinas J., Fought A.J., Topol E.J. (2014). Comparison of 24-hour Holter monitoring with 14-day novel adhesive patch electrocardiographic monitoring. Am. J. Med..

[20] Bayoumy K., Gaber M., Elshafeey A., Mhaimeed O., Dineen E.H., Marvel F.A., Martin S.S., Muse E.D., Turakhia M.P., Tarakji K.G. (2021). Smart wearable devices in cardiovascular care: Where we are and how to move forward. Nat. Rev. Cardiol..

[21] Giebel G.D., Gissel C. (2019). Accuracy of mHealth devices for atrial fibrillation screening: Systematic review. JMIR mHealth uHealth.

[22] Yang T.Y., Huang L., Malwade S., Hsu C.Y., Chen Y.C. (2021). Diagnostic accuracy of ambulatory devices in detecting atrial fibrillation: Systematic review and meta-analysis. JMIR Mhealth Uhealth.

[23] Zhu H., Pan Y., Wu F., Huan R. (2019). Optimized electrode locations for wearable single-lead ECG monitoring devices: A case study using WFEES modules based on the LANS method. Sensors.

[24] Bland J.M., Altman D.G. (1986). Statistical methods for assessing agreement between two methods of clinical measurement. Lancet.

[25] Stockl D., Rodriguez Cabaleiro D., Van Uytfanghe K., Thienpont L.M. (2004). Interpreting method comparison studies by use of the Bland-Altman plot: Reflecting the importance of sample size by incorporating confidence limits and predefined error limits in the graphic. Clin. Chem..

[26] Bansal A., Joshi R. (2018). Portable out-of-hospital electrocardiography: A review of current technologies. J. Arrhythm..

[27] Al-Alusi M.A., Ding E., McManus D.D., Lubitz S.A. (2019). Wearing your heart on your sleeve: The future of cardiac rhythm monitoring. Curr. Cardiol. Rep..

[28] Mehta D.D., Nazir N.T., Trohman R.G., Volgman A.S. (2015). Single-lead portable ECG devices: Perceptions and clinical accuracy compared to conventional cardiac monitoring. J. Electrocardiol..

[29] Desteghe L., Raymaekers Z., Lutin M., Vijgen J., Dilling-Boer D., Koopman P., Schurmans J., Vanduynhoven P., Dendale P., Heidbuchel H. (2017). Performance of handheld electrocardiogram devices to detect atrial fibrillation in a cardiology and geriatric ward setting. Europace.

[30] Wong K.C., Klimis H., Lowres N., von Huben A., Marschner S., Chow C.K. (2020). Diagnostic accuracy of handheld electrocardiogram devices in detecting atrial fibrillation in adults in community versus hospital settings: A systematic review and meta-analysis. Heart.

[31] Pevnick J.M., Birkeland K., Zimmer R., Elad Y., Kedan I. (2018). Wearable technology for cardiology: An update and framework for the future. Trends Cardiovasc. Med..

[32] Ackermans P.A., Solosko T.A., Spencer E.C., Gehman S.E., Nammi K., Engel J., Russell J.K. (2012). A user-friendly integrated monitor-adhesive patch for long-term ambulatory electrocardiogram monitoring. J. Electrocardiol..

[33] Ramkumar S., Nerlekar N., D’Souza D., Pol D.J., Kalman J.M., Marwick T.H. (2018). Atrial fibrillation detection using single lead portable electrocardiographic monitoring: A systematic review and meta-analysis. BMJ Open.

[34] Cheung C.C., Kerr C.R., Krahn A.D. (2014). Comparing 14-day adhesive patch with 24-h Holter monitoring. Future Cardiol..

[35] Jacobs M.S., Kaasenbrood F., Postma M.J., van Hulst M., Tieleman R.G. (2018). Cost-effectiveness of screening for atrial fibrillation in primary care with a handheld, single-lead electrocardiogram device in the Netherlands. Europace.

